# School-Based Surveillance of Respiratory Pathogens on “High-Touch” Surfaces

**DOI:** 10.3389/fped.2021.686386

**Published:** 2021-06-22

**Authors:** Alastair F. Murray, Anne Emanuels, Caitlin Wolf, Nicholas Franko, Lea Starita, Janet A. Englund, Helen Y. Chu

**Affiliations:** ^1^Department of Pediatrics, University of Washington, Seattle, WA, United States; ^2^Division of Allergy and Infectious Diseases, University of Washington, Seattle, WA, United States; ^3^Department of Genome Sciences, University of Washington, Seattle, WA, United States; ^4^Infectious Disease and Virology, Seattle Children's Hospital, Seattle, WA, United States

**Keywords:** respiratory virus, school health, influenza, rhinovirus, viral surveillance, fomite surface, adenovirus

## Abstract

In order to assess the presence of respiratory pathogens on “high-touch” surfaces and inform sanitation practices at schools, pre-selected surfaces in elementary schools in Seattle, WA, USA were sampled weekly and tested by RT-PCR for 25 viral respiratory pathogens (including SARS-CoV-2 retrospectively) and *S. pneumoniae* during 2019–2020 winter respiratory illness season. Viral pathogens (rhinovirus, adenovirus, influenza) known to cause respiratory illness were detected on commonly touched surfaces, especially wooden surfaces, and matched the patterns of circulating virus in the community.

## Introduction

The emergence of SARS-CoV-2 has further underscored the importance of detecting and eliminating respiratory pathogens in high-risk congregate settings. The density and proximity of children within school and childcare facilities provides a ready environment for infectious disease transmission (1, 2). Previous environmental surveillance studies for respiratory pathogens have used bioaerosol sampling and surface swabbing techniques to detect spread of respiratory viruses (3, 4). To identify sources of pathogen transmission in the school environment, we sampled “high-touch” surfaces in elementary schools in Seattle, WA, USA and performed weekly air sampling during the winter respiratory illness season of 2019–2020.

## Methods

**Figure 1:**
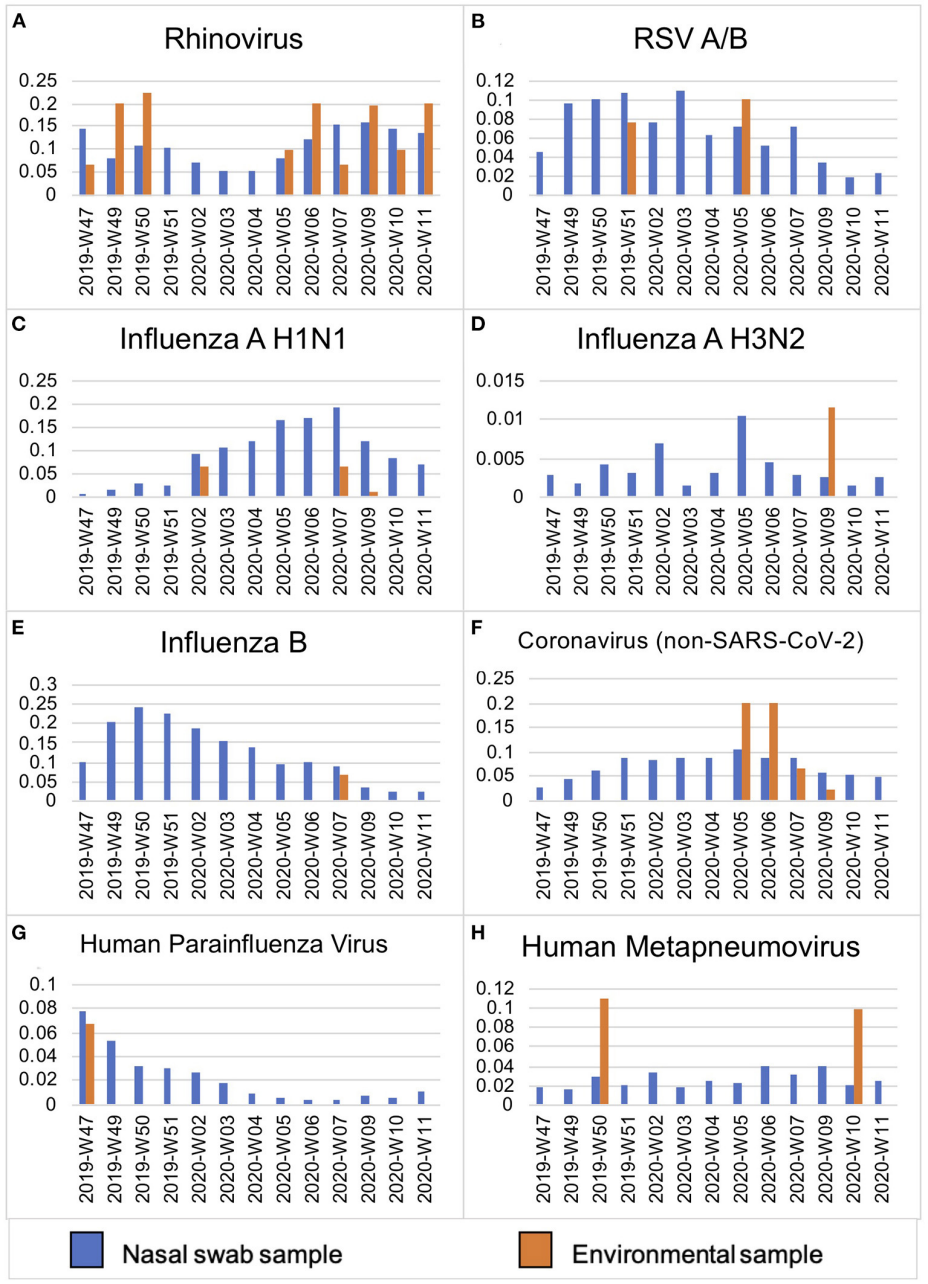
Viral prevalence by week in Seattle, WA from November 2019–March 2020 in symptomatic individuals as compared to environmental surface swabs in schools. Prevalence (y-axis) of viral pathogens **(A–H)** detected in de-identified residual samples from hospitalized patients and prospective mid-turbinate swabs from symptomatic individuals in Seattle, WA, USA (*n* = 12,458) as compared to contemporaneous environmental surface swab sampling in Seattle public schools (*n* = 208) between November 2019–March 2020 (by week, x-axis).

Surface swabs were collected weekly at three public elementary schools in Seattle from November 2019 to March 2020. High-touch plastic, wood and metal surfaces including door and fountain handles and handrails were pre-selected and sampled weekly by thoroughly wiping with a synthetic polyester swab in the same time window (5). Additionally, intensive twice daily sampling during the last week of February 2020 was conducted in two schools in mornings and prior to dismissal. Scheduled school cleaning typically took place daily in the afternoons after dismissal. Bioaerosol samples from a cafeteria and hallway were collected during a weekly 90-min period using the SKC QuickTake 30 Air Pump (5). All samples were tested for 25 respiratory viruses including SARS-CoV-2 and *S. pneumoniae* by multiplex TaqMan real-time PCR on the OpenArray platform (Thermo). Cycle relative threshold (Crt) values were measured for each pathogen and for ribonuclease (RNAse) P, a marker of human cellular material detected on swabs. Prevalence of pathogens was compared to city-wide prevalence of viral infections captured by a related, contemporaneous study (Figure 1) (5).

## Results

Of samples collected at three schools, 49 (34%) of 208 total samples tested positive for one or more viruses, most commonly human rhinovirus (hRV) (28/208, 13%) and adenovirus (14/208, 6.7%) (Table 1). Overall, 65% of samples were positive for *S. pneumoniae*. No SARS-CoV-2 was detected on any of the samples tested. Overall mean RNAse P Crt value was 25 (s.d. 2.9) with no significant difference by surface material type (plastic, wood or metal). There was no significant difference in viral and bacterial Crt values across surface material type, although wooden surfaces had the highest quantity of viral detections (average, 1.3 viral detections per sample collected) followed by metal (average, 0.2 viral detections per sample). No virus was detected through bioaerosol sampling. During the week of twice daily intensive sampling, no virus was persistently detected on a surface for more than two consecutive samples. More viruses were found during morning compared to afternoon sampling, but not significantly so (*t*-test, *p* = 0.24).

**Table 1:** Sites, surfaces, and pathogens detected between 13 surfaces sampled across three elementary schools in Seattle, WA.

			**Pathogen**									
			***n*** **(%)**									
	**Samples collected *(n)***	**Material**	**Flu A/B**	**RSV A/B**	**hPIV I-IV**	**hMPV**	**Human rhinovirus**	**hCoV**	**AdV**	**hBoV**	**Enterovirus**	***S. pneumo***
**School A**												
Door (front entrance)	20	Metal	0	1 (5.0%)	0	0	1 (5.0%)	1 (5.0%)	0	2 (10%)	0	5 (25%)
Handrail (to classroom)	21	Wood	1 (4.8%)	1 (4.8%)	1 (4.8%)	1 (4.8%)	11 (52%)	3 (14%)	4 (19%)	5 (24%)	4 (19%)	21 (100%)
Water fountain handle	21	Metal	0	0	0	0	4 (19%)	0	1 (4.8%)	0	0	17 (81%)
Door (to playground)	21	Metal	0	0	0	0	2 (9.5%)	0	2 (9.5%)	2 (9.5%)	0	14 (67%)
**School B**												
Door (front entrance)	22	Metal	0	0	0	0	0	0	1 (4.5%)	0	0	12 (55%)
Door (gymnasium)	21	Metal	1 (4.8%)	0	0	1 (4.8%)	5 (24%)	2 (9.5%)	2 (9.5%)	0	0	15 (71%)
Table surface (cafeteria)	21	Plastic	1 (4.8%)	0	0	0	1 (4.8%)	1 (4.8%)	0	0	0	10 (48%)
Door (to playground)	21	Metal	1 (4.8%)	0	0	0	1 (4.8%)	0	1 (4.8%)	1 (4.8%)	0	13 (62%)
**School C**												
Door (front entrance)	8	Metal	0	0	0	0	0	0	0	0	0	4 (50%)
Pinpad (cafeteria)	8	Metal	0	0	0	0	0	0	0	0	0	4 (50%)
Door (to playground)	8	Metal	0	0	0	0	0	0	0	0	0	7 (88%)
Water fountain handle	8	Metal	0	0	0	0	0	0	1 (13%)	1 (13%)	0	5 (63%)
Handrail (to lower level)	8	Wood	1 (13%)	0	0	0	3 (38%)	0	2 (25%)	1 (13%)	1 (13%)	8 (100%)

*hPIV, human parainfluenza virus; hMPV, human metapneumovirus; hCoV, human coronavirus (non-SARS CoV-2); AdV, adenovirus; hBoV, human bocavirus*.

## Discussion

At the onset of the SARS-CoV-2 pandemic, restrictions were placed on in-person learning in the United States to help decrease viral transmission. Given the prolonged disruption to childhood education, there is an increasing need for evidence to guide safe school reopening policies and inform sanitation practices (6, 7). This school-based surveillance study conducted around the time of United States community transmission of SARS-CoV-2 (November 2019–March 2020) and before school closures, shows that respiratory pathogens known to cause illness were detected on commonly touched surfaces. Risks posed from fomite transmission vs. aerosol transmission are pathogen-specific (8–10).

Nasopharyngeal colonization with *S. pneumoniae* has been well-described in the pediatric population, and in this study *S. pneumoniae* was near ubiquitous on surfaces. Previous studies have demonstrated the fomite potential of *S. pneumoniae*, through tolerance of desiccation and the potential to remain infectious on rehydration (11). It is unlikely that the presence of *S. pneumoniae* on these surfaces resulted in clinical illness, both in terms of quality of inoculum and serotype in the post-pneumococcal vaccine era (12).

Rhinovirus and adenovirus, both non-enveloped viruses known to persist under harsh environmental conditions, were also the most commonly detected on surfaces at schools in this study (13). It is unclear if the quality or viral load of the inoculum was sufficient to contribute to direct spread of disease, as we did not culture for pathogens. However, the viruses found on surfaces at schools did mirror the prevalence pattern of viral positives in the community from individuals with symptomatic respiratory illness (5) (Figure 1). This finding demonstrates how environmental surface sampling could serve as a non-invasive surveillance tool to detect human viral circulation within a larger community.

Considering how this data may inform school sanitation practices, we found that, across three elementary schools, high-touch wooden surfaces had the highest number of viral pathogen detections. Overall, viral presence on surfaces was transient and did not persist for longer than 1 day (two consecutive samplings) during the intensive week of sampling, either as result of natural degradation or efficacy of standard cleaning practices (14). As schools continue to re-open, frequent targeted cleaning of high-touch surfaces in schools, particularly wooden surfaces, can complement other measures to reduce potential fomite transmission of perennial respiratory pathogens in the context of the global SARS-CoV-2 pandemic.

## Data Availability Statement

The raw data supporting the conclusions of this article will be made available by the authors, without undue reservation.

## Author Contributions

AM drafted the manuscript and performed data analysis with assistance from NF and AE. AE and CW contributed to methodology and oversight. LS, HC, and JE obtained funding and conceptualized the study in addition to providing oversight. LS assisted with investigation and data collection. All authors have reviewed and commented on the manuscript.

## Conflict of Interest

HC has received research support from Cepheid, and consults for Merck, Pfizer, Gates Foundation, and Ellume. JE has received research support from GlaxoSmithKline, Merck, Pfizer, and AstraZeneca and personal fees from Sanofi Pasteur and Meissa Vaccines. The remaining authors declare that the research was conducted in the absence of any commercial or financial relationships that could be construed as a potential conflict of interest.
